# Guidelines needed for the use of AI in the preparation or review of IRB, IBC, and IACUC applications

**DOI:** 10.1080/08989621.2025.2612564

**Published:** 2026-01-08

**Authors:** Mohammad Hosseini, Daniel Eisenman, James Riddle, Stephanie Pyle, Anju Peters, Nichelle Cobb, And Kristi Holmes

**Affiliations:** a Department of Preventive Medicine, Northwestern University Feinberg School of Medicine, Chicago, IL, USA; b Galter Health Sciences Library and Learning Center, Northwestern University Feinberg School of Medicine, Chicago, IL, USA; c Global Review Services, Advarra, Columbia, MD, USA; d Department of Medicine, Northwestern University Feinberg School of Medicine, Chicago, IL, USA; e Association for the Accreditation of Human Research Protection Programs, Washington DC, USA

**Keywords:** Artificial intelligence, Ethics, IRB, IBC, IACUC

## Abstract

Three oversight bodies review research proposals to help ensure the safe and responsible conduct of biomedical research, each focusing on unique aspects of research ethics: institutional review boards (IRBs), institutional biosafety committees (IBCs), and institutional animal care and use committees (IACUCs). The role of artificial intelligence (AI) in research oversight is rapidly expanding, specifically when preparing and reviewing applications. Although using AI may reduce administrative costs and burdens, it also may create new concerns since AI tools can make mistakes of fact and reasoning, and are susceptible to bias. Furthermore, outsourcing ethical planning and oversight of research to AI could compromise ethical understanding. Although the arguments for/against using AI in preparation or review of IRB, IBC, or IACUC differ fundamentally from those concerning AI use in manuscript writing/peer review, currently there is minimal guidance about the responsible use of AI in research oversight from government agencies, professional organizations, universities, hospitals, and other entities that conduct research. We argue that 1) to minimize the risks of using AI in research oversight, additional guidance is urgently needed; and 2) humans must always be the final decider because ethical planning and oversight involve value judgments that should not be outsourced to AI.

## Introduction

Three committees are responsible for overseeing different aspects of the ethical conduct of research:

- Institutional review boards (IRBs) are responsible for helping to protect the rights and welfare of human research participants.
- Institutional biosafety committees (IBCs) oversee the use of engineered genetic material (e.g., recombinant or synthetic nucleic acid molecules) and help protect researchers, the community, and environment from risks related to genetically modified organisms and dangerous biological agents.
- Institutional animal care and use committees (IACUCs) are charged with reviewing research and teaching activities involving animals to ensure their ethical use, humane treatment, and the highest standards of care.

Together, these three committees are sometimes referred to as the *Three I’s*.

Although most research professionals who interact with the Three I’s recognize the importance of independent oversight, many criticize the administrative burdens associated with the research review process, such as the inordinate amount of time and effort spent on writing protocols, preparing applications for the oversight committees, annual reports, and other documentation that must be submitted for these committees’ review (Schneider 2015; Silberman and Kahn 2011).

On the other hand, committee members also are burdened by the time and effort they spend reading study documents and ensuring that research proposals comply with regulations, institutional policies, and ethical guidelines (Klitzman 2015). The time spent by panel members, chairs, coordinators, and staff represents a substantive cost to conducting research. Funding allocated to researchers and institutions for these oversight activities is declining in numerous countries, including the United States and the Netherlands (Higher Education Press Agency 2025; Oliver 2025).

According to a 2003 estimate, depending on the volume of applications, universities may spend up to $770,674 per year (about $1.4 million in 2025 US dollars) to operate an IRB (Wagner et al. 2003). Factors involved in the costs of running these committees are discussed further by others (Speckman et al. 2007; Sugarman et al. 2005). According to a 2023 report published by the Association for Accreditation of Human Research Protection Programs (AAHRPP), depending on the number of active studies and staff, the IRB budget could vary between $294,916 and $2,517,398 (AAHRPP 2025). Our personal communication with a top-tier US-based academic center suggests that the annual costs of running an institutionally based IRB in 2024 were around $4.5 million. While such figures are not as readily available for administering an institutional IBC or IACUC, anecdotal evidence suggests these oversight bodies require similarly significant investments to maintain.

As a result of recent advances in artificial intelligence (AI), it may now be possible to reduce some of the administrative costs and burdens associated with the Three I’s. An increasingly available array of AI tools equipped with large language models (LLMs) could assist researchers in drafting and refining applications, protocols, and other documents submitted to these committees. AI tools also could assist committee members and administrators in reviewing submitted applications by, for example, summarizing, identifying ethical and regulatory issues that are not adequately addressed, and making suggestions for rewriting sections/forms for clarity and readability. Even the US Food and Drug Administration (FDA) has announced that it had completed a program to pilot the use of AI to assist with review of product applications (Food and Drug Administration 2025).

This paper aims to explore the ethical implications of using AI in preparing or reviewing applications for the Three I’s. Although some concerns apply across all three committee contexts (e.g., the risk of bias or errors), we deliberately keep these contexts separate to reflect the specific challenges others have reported and to explain these in detail, so that readers can engage with the section most relevant to their expertise, research focus, and/or professional role.

## Special considerations for IRBs

IRBs are a vital institutional actor for research oversight and play a crucial role in upholding the ethical and safe conduct of research. Preparing an IRB application is a detailed and time-consuming process, and some researchers may consider using AI tools for this purpose. Although this has not been independently validated, developers of AI tools like the “IRB Draft Generator” suggest that such tools can create substantial portions of an IRB application (Sugarman et al. 2005). Upon receiving four components (i.e., brief research description, study hypothesis, inclusion/exclusion criteria, and study design type), the tool reportedly performs a literature search using PubMed and generates a draft protocol document. AI is also reported to create informed consent forms that could be more readable than those written by humans (Decker et al. 2023). Other AI-driven applications built on ChatGPT (e.g., IRB Guide, IRB Advisor) are reportedly fine-tuned to help researchers prepare their IRB applications. However, in a preliminary report of a systematic review of these applications (*n* = 42), Kwon and colleagues noted that only four had been specifically trained for IRB-related purposes (one of those was only trained with a single IRB-approved informed consent form). Only five of the assessed applications could describe an IRB’s role, the types of research an IRB typically reviews, as well as standard informed consent requirements (Kwon et al. 2024). Empirical assessments like this demonstrate that when researchers rely on currently available AI tools to write their applications, they may not be providing the IRB with enough accurate information to permit a thorough review that meets regulatory requirements.

AI has also been explored as a tool to review IRB applications. For example, Mann and colleagues suggested developing LLMs tailored for IRB review of research applications (Mann et al. 2025). These systems could be fine-tuned using IRB-specific documents, past review decisions, and local policies. Authors suggested further enhancing these tools by providing access to institutionally relevant data and techniques that improve accuracy and relevance of reviews, such as retrieval-augmented generation (RAG). Authors noted that such systems could screen IRB submissions for completeness, warn of ethical red flags, generate preliminary reviews, and evaluate consistency with past committee decisions or institutional policies. While acknowledging the risks of over-reliance, opacity, and potential biases, Mann and colleagues argued that properly audited LLMs with human-in-the-loop could improve IRB review processes.

Separately, Sridharan and Sivaramakrishnan assessed the decision-making capabilities of ChatGPT, Poe Assistant, and Google Bard (now Gemini) by asking them to consider ten IRB case scenarios (Sridharan and Sivaramakrishnan 2024). The authors reported that AI platforms identified areas for improvement, suggested corrective actions, highlighted conflicts of interest, and recognized vulnerable populations. However, the quality of these assessments was not evaluated by authors. Furthermore, none of the AI tools Sridharan and Sivaramakrishnan examined could consistently address quorum requirements, which are essential for ensuring diverse IRB representation and appropriate review of research. Similarly, the AI tools provided only incomplete or inconsistent risk assessments and poor site monitoring suggestions which may hinder the broader functions of Human Research Protection Programs (HRPPs), including but not limited to post-approval monitoring and oversight mechanisms used to identify potential performance issues such as excessive noncompliance or unanticipated problems that could affect the rights and welfare of research participants. Additionally, some custom GPTs within the ChatGPT environment (e.g., IRB Reviewer Helper) claim to support IRB application reviews. But thus far, no published report has assessed the validity or reliability of these tools for review purposes.

Despite these shortcomings, Sridharan and Sivaramakrishnan suggested that a validated and locally hosted AI could significantly enhance IRB review efficiency. With “locally hosted AI,” authors seem to refer to systems deployed within an institution’s own secure infrastructure, which allows control over data storage and access. Such a locally hosted AI could help ensure compliance with local requirements and offer more control over outputs. Although local AI instances offer better privacy and customization, they require inhouse technical expertise, computational resources, and maintenance, all of which can undo savings initially promised by AI integration.

### Challenges of using AI for preparing and reviewing IRB applications

In terms of using AI to prepare IRB applications, perhaps the most significant challenge is the introduction of factual, procedural, or interpretive errors in the documents submitted for review. For example, AI-generated summaries of drug safety data, dosing, or procedural protocols might contain errors and miss or downplay risks, which could potentially lead to the research team (and subsequently the IRB) coming to erroneous conclusions and inadequate safeguards for human participants. The same is true about biases, especially when AI is used to conduct a review of the literature for preparing IRB applications. If unrecognized by the research team or IRB, biased review of the literature and inaccurate or incomplete assessment of extant knowledge could lead to erroneous conclusions regarding the involved risks and the appropriateness of the research.

It is true that human researchers make mistakes too, and may have produced erroneous or biased reviews in the past, e.g., including retracted articles in reviews (Audisio et al. 2022). However, AI-generated reviews used in IRB applications pose new risks, including:

- AI might not have access to or analyze the full texts of all relevant pieces and could make conclusions based only on publicly available abstracts, which can be biased and provide an incomplete representation of the entire study (Duyx et al. 2019) or cite hallucinated and non-existing studies;
- AI searches are not always reproducible and could yield different results depending on the prompt (or chain of prompts) used (Hosseini and Horbach 2023);
- AI’s (sometimes) inappropriate cultural assumptions and social orientations, based on limited and/or biased training data, could increase the risk of bias and discrimination against certain participant populations;
- AI interpretations of available and related studies can be one-sided or entirely wrong (Lu, Song, and Zhang 2025).

Confidentiality concerns are serious too. IRB applications may contain detailed descriptions about participating cohorts which, to protect those cohorts’ identities, may never be published in a resulting manuscript. Using AI, especially third-party or commercial platforms, could introduce unacceptable risks to data security and legal compliance. For example, IRB applications might include demographic details, geographical whereabouts, and affiliation information about participants (e.g., the name of their institution or tribal community), but the resulting manuscript would omit this information in order to protect participants. Submitting IRB applications to AI tools with such information included could violate researcher obligations to protect participants from harm or exposure. Additionally, some AI systems might lack transparency about data retention, usage and sharing practices (Hosseini et al. 2025). Without clear contractual guarantees or compliance frameworks in place, IRBs cannot ensure that uploaded content is not stored by the system’s developer, used to train future models, or accessed by unauthorized parties.

## Special considerations for IBCs

IBCs are charged with reviewing research involving engineered genetic materials and conducting a thorough risk assessment to ensure the requisite risk mitigation plan will adequately contain potential risks. In the US, IBCs ensure compliance with the National Institutes of Health (NIH) Guidelines for Research Involving Recombinant or Synthetic Nucleic Acid Molecules (NIH Guidelines 2024). This includes assessing a site’s risk mitigation plan to ensure it includes training and supervision of research personnel; assessing the adequacy of research facilities, safety equipment, and personal protective equipment (PPE); and reviewing a site’s spill and incident response plans. According to NIH Guidelines, the PI is responsible for conducting the initial risk assessment for the proposed research, including determining the required levels of physical and biological containment. An IBC must be composed of at least five committee members who collectively possess the experience and expertise needed to assess research safety by identifying potential risks to researchers, public health, or the environment. The committee’s membership must include at least two local unaffiliated community members who represent the interests of the community and the environment around the research site.

AI tools can be used to prepare or review an IBC application. For example, AI may be beneficial to investigators to help ensure inclusion of the necessary sections required by an IBC. According to some preliminary studies, AI models have demonstrated the ability to troubleshoot lab procedures and protocols involving microorganisms at a level that can exceed experienced microbiologists (Chow 2025; Göttinga et al. 2025). Furthermore, AI can be used to review and triage applications submitted to an IBC. However, given the specific purpose of an IBC submission, and unique responsibilities of IBC reviewers, challenges of using AI in this context are different from those discussed about IRBs.

### Challenges of using AI for preparing and reviewing IBC applications

AI is currently unable to comprehensively assess the risks related to proposed procedures, expertise of the research personnel, available facilities, safety equipment, and PPE. *In vivo* research, including animal models and clinical trials, introduces additional layers of complexity, such as concerns about subjects’ welfare (human or animal), investigational product containment, and potential exposure to individuals or animals not directly involved in the study. This underscores the broader question of whether AI should play any role in the ethical planning and oversight of such experiments. AI can enable unskilled individuals or a novice microbiologist to draft applications for modifying microorganisms without the experience or expertise needed to assess whether the modified microbe might pose a risk to themselves or others. AI can also enable individuals with nefarious intent to skirt existing oversight systems and perform experiments they otherwise could not.

In terms of reviewing IBC applications, different institutions may have different research capabilities, levels of funding, and institutional risk tolerances. Accordingly, a risk mitigation plan suitable for a research project at one institution may be completely inappropriate for another. Given these nuances involved in IBC review, an algorithm’s formulaic approach would omit key components of a comprehensive risk assessment and review of the risk mitigation plan.

That said, AI tools could be utilized by an IBC to assist committee members and administrators in comparing projects to ensure consistent determinations. Validated AI tools trained with high quality data, protocols, and publications can also serve as a tool to compare proposed genetic modifications to those in the published literature to ensure real-world data is included in IBC risk assessments.

We believe that given the importance of ensuring safety and security for research, the roles of risk assessment, risk mitigation and ethical judgments made by IBCs cannot be delegated to AI. Biosafety risk assessments and risk mitigation plans vary by study and research location and require experienced individuals to assess whether protections are appropriate in a specific context. The role of the local unaffiliated community members in representing their community and advocating for their environmental challenges cannot be replaced either. AI can assist IBCs in performing reviews, for example, by identifying similar applications that were previously reviewed to aid in producing consistent determinations. However, AI’s limitations prevent it from unilaterally or autonomously conducting a thorough review of research risks and adequately planning or suggesting mitigation strategies.

Within the purview of IBC review and oversight is the safe conduct of experimental manipulations and genetic modification of infectious agents. Such oversight extends to AI controlled laboratory robots performing experimental procedures. Progress is being made toward developing automated self-driving labs (SDLs), with data-driven experiment planning and automated experiment execution by integrating robotics and laboratory equipment (Abolhasani and Kumacheva 2023). The next generation is represented by AI agents like ORGANA, a robot designed to conduct laboratory experiments (Darvish et al. 2025). ORGANA uses LLMs to interact with researchers and receive experimental parameters such as the experimental design and objectives prior to autonomously carrying out an experiment and compiling a report. Similarly, Coscientist, is an AI system powered by LLMs that can autonomously design, plan and perform certain experiments (Boiko et al. 2023). Should IBCs allow AI to be involved in drafting or reviewing proposals that involve SDLs or AI agents performing genetic manipulations of pathogenic microorganisms? What if the AI model is hacked, corrupted or the reviewing AI is positively biased toward other automated systems and delivers unduly positive evaluations? In such a scenario, biosafety and cyber-security will be compromised with potentially catastrophic consequences.

Questions about AI use within IBC workflows and laboratory research arise at a time when the US government is modernizing NIH Guidelines to “keep pace with the evolving risks posed by today’s rapidly advancing science and technology” and “to ensure that 21^st^ century biosafety oversight meets the research needs of both today and tomorrow” (National Institutes of Health 2025). The federal government is specifically concerned about safety and security in research utilizing genetically modified biologics. The 5 May 2025, presidential Executive Order (EO) on Improving the Safety and Security of Biological Research calls for revising or replacing the existing *United States Government Policy for Oversight of Dual Use Research of Concern and Pathogens with Enhanced Pandemic Potential* (The White House 2025). This EO highlighted Gain of Function research, which confers genetic modifications to microorganisms and can result in potentially dangerous pathogens: “Dangerous gain-of-function research on biological agents and pathogens has the potential to significantly endanger the lives of American citizens. If left unrestricted, its effects can include widespread mortality, an impaired public health system, disrupted American livelihoods, and diminished economic and national security.” The May 2025 EO defines Dangerous Gain-of-Function Research as “scientific research on an infectious agent or toxin with the potential to cause disease by enhancing its pathogenicity or increasing its transmissibility. Covered research activities are those that could result in significant societal consequences.” Examples of experimental manipulations worrying the government include modifying microbes to disrupt beneficial immunological responses or the effectiveness of immunizations, conferring resistance to clinically or agriculturally useful prophylactics or therapeutics, and expanding the host range so the organism can infect hosts it previously could not. Resurrecting extinct agents or toxins, such as smallpox is also a concern. Other experts have called for built-in biosecurity safeguards for generative AI tools (Wang et al. 2025).

Implementation of various AI safeguards could mitigate the risk of utilizing AI in biological research. For example, imposing a requirement for watermarking of AI-generated biological designs would allow for identification of these constructs. We believe that potential new regulations could mandate developers of AI tools to avoid generating knowingly harmful protocols, proteins, or genetic manipulations, report such attempts and/or propose sanctions for non-adherence. Furthermore, virulence factors or other potentially harmful gene sequences from high consequence infectious agents or toxins could be restricted, akin to restrictions already in place for vendors synthesizing DNA.

## Special considerations for IACUCs

IACUCs occupy a distinct regulatory and ethical role within the research oversight ecosystem. Their responsibility for ensuring the humane and responsible care and use of animals in research, testing, and teaching requires a deliberative, context-sensitive assessment process. IACUC protocols must include detailed justifications for the use of specific species and animal numbers, outline procedures for pain mitigation, and demonstrate adherence to the ethical principles of replacement, reduction, and refinement, so-called 3Rs (OLAW 2020; USDA 2021). These assessments are rooted in regulatory compliance and in ethical reasoning that considers animal welfare in light of scientific necessity. This complexity is why the *Guide for the Care and Use of Laboratory Animals*, published by the National Academies Press, calls for multidisciplinary review, ongoing protocol oversight, and institutional accountability (National Research Council 2011).

As with IRBs and IBCs, researchers and administrators may be exploring AI tools to help prepare or review IACUC protocols (e.g., Protocol Assistant Chat GPT and Modern Vivo). While it is conceivable that generative AI could assist with writing boilerplate language regarding protections for a specific animal or enhancing document formatting, we believe that AI’s utility ends there for IACUC activities.

In addition to errors and biases (more on this shortly), AI currently lacks the capability to interpret the appropriateness of humane endpoints, the adequacy of species-specific enrichment, or the implications of serial procedures (including multiple major surgeries). Indeed, AI cannot substitute for veterinary expertise nor ethical judgment in weighing scientific objectives against the potential for animal distress. Similar to IBC and IRB review, relying heavily on AI for assessing risks and harms or leaving IACUC review solely to AI currently yields more risk than benefits.

### Challenges of using AI for preparing and reviewing IACUC applications

Recent empirical research confirms the complexity and variability of IACUC decision-making. In a scoping review of studies on how Animal Ethics Committees and IACUCs make decisions, Milford and colleagues found that harm-benefit analyses, assessments of the 3Rs, and deliberative judgments about justification and refinement vary widely and are influenced by committee composition, member experience, and contextual interpretation of institutional norms (Milford et al. 2025). The review found no standardized method for evaluating ethical acceptability across studies, reinforcing that these judgments require human values and interpersonal negotiation, and cannot be replicated with machine learning or algorithmic pattern matching. In a recent attempt, researchers at University of Pittsburgh have tested with OpenAI and Gemini for prescreening proposals to identify common errors submitted to IACUC (Vasquez et al. 2025). The authors note that all protocols are reviewed by human experts after this prescreening stage to detect more complex “regulatory, research, clinical, and ethical issues that may not have been addressed through the initial errordetection process.” However, they also stress that interpreting and comparing tabular information, such as those related to “proper identification of pain and distress classifications, and classification of drugs and chemicals” were challenging for AI, and on average, these systems achieved precision scores at 80% and higher. Such findings underscore the limitations of AI in replicating the required depth and nuance of ethical review in oversight of animal research, and perhaps, other research oversight mechanisms.

Moreover, AI use within IACUC workflows introduces unique security and confidentiality concerns. IACUC protocols often contain detailed information about specific animal housing locations, room numbers, species and strain data, personnel assignments, and procedural timing. This information is typically subject to institutional controls and security and confidentiality guidance provided by the Office of Laboratory Animal Welfare (OLAW 2023). Even when an AI system is deployed within a so-called “walled garden” or internal university instance (where data remains local and is not used for broader training), sensitive data points could be cached, used for model refinement, or inadvertently exposed through system logs and data leaks. These risks are not theoretical. Institutions increasingly face heightened scrutiny and active threats related to animal research programs (Bailey, Rich, and Bennett 2010; Goode 2025; McLeod and Hartley 2017; The Center for Animal Law Studies 2024). A breach involving IACUC data could compromise researcher safety, disrupt facility operations, and/or expose institutions to public backlash and legal liabilities.

As with IRB and IBC, ethical judgments made by IACUCs cannot be delegated to AI. The decisions made by these committees are neither mechanical nor transactional; rather, they are grounded in responsibility for the welfare of living beings entrusted to research institutions. While AI may provide support for routine, basic administrative tasks, it cannot replace the empathetic, experience-informed human deliberation that IACUC reviews demand.

## AI’s limitations for Three I’s and the need for guidance

Although using AI to assist with research planning and oversight may reduce administrative costs and burdens, it also raises additional concerns: these tools can and will make mistakes of fact and reasoning, and they are susceptible to bias (Resnik and Hosseini 2024). Compounding this problem is the possibility that researchers and reviewers who use these tools may be under the false impression that the AI generated content or review is accurate and thus, does not need to be thoroughly validated and verified by humans.

Currently, manual validation and verification methods are still the most reliable forms of vetting AI-assisted work. However, since such work is tedious and extremely time-consuming, it could undo potential productivity gains promised by AI. Further, the Three I’s contexts are built on trust, and committees rely on researchers to provide accurate information, for example, about drug dosing or potential risks as they are reflected in the literature. If researchers submit inaccurate proposals co-written by AI, the reviewing committee (with or without AI support) may then make inaccurate assessments. Such assessments based on incomplete or inaccurate information synthesized/generated by AI could lead to determinations that do not provide or promote the most optimal protections for research subjects. Furthermore, if/when AI models developed by the same company (e.g., OpenAI, Google) are used in both preparation and reviewing of applications, the potential for biases and creating overly favorable feedback loops could increase. Such echo chambers could limit diversity of perspectives and lead to diminished critical scrutiny of high-risk or novel proposals.

Challenges about the responsible use of AI in the Three I’s are not currently addressed in guidance from research organizations (e.g., universities and hospitals) or authoritative bodies (e.g., government agencies or professional organizations). Thus, to minimize the risks related to using AI in the Three I’s, guidance is urgently needed (Resnik, Hosseini, and Cobb 2024). Since there is already guidance for using AI to write and review scholarly publications (Resnik and Hosseini 2025), some might question the indispensability of specific guidance for the use of AI to prepare or review Three I’s submissions. However, such objections overlook the unique considerations involved in the Three I’s, three of which are discussed below.

First, mistakes that occur during research oversight can lead directly to sustained significant harm to human participants, animals, the environment, or public health. For example, federal investigations determined that Ellen Roche’s 1998 death in a trial conducted at Johns Hopkins University was due, in part, to inadequate review of the background literature on the risks associated with the study drug. The investigator and the IRB were both partly blamed for this tragic mistake (Resnik 2024). In another example, during the 2000 Oklahoma Melanoma Vaccine Trial, the IRB failed to ensure compliance, contamination checks, and proper transfer, injection, and dose tracking that resulted in 26 deaths (Weiss and Nelson 2000). Given the concerns outlined in this paper, conducting a review for a Three I’s submission with AI could potentially increase the likelihood of such tragedies. When AI assists with writing manuscripts, the potential errors it introduces can be scrutinized by the scientific community and, if necessary, will result in retractions such as the case of an AI-generated image of a rat with unusual genitals (Pearson 2024) or the retraction of 129 papers by *Neurosurgical Review* (Retraction Watch 2025). In contrast, since protocols submitted to the Three I’s are not openly shared nor published, they cannot be easily critiqued by peers or readers. Subsequently, if errors (introduced by AI or due to negligence) go unnoticed during the review process, they could potentially lead to serious harms which might remain unnoticed unless an investigation is initiated, but they sometimes may never be found even after the study has been completed. For instance, AI-generated summaries of drug safety data or procedural protocols might miss or downplay risks, potentially leading to inadequate safeguards for human participants, or research animals. In other words, because Three I’s applications and review determinations are not published publicly, any misjudgment in the review process will likely not be caught by the wider community until the potential ramifications are experienced in the form of physical, psychological, or legal harm to research participants, researchers, or the environment.

Second, research has shown that even bespoke AI models might fail to adhere to regulatory logic or context-specific requirements (Kwon et al. 2024). AI can generate plausible-sounding text, but it cannot *understand* what makes a protocol ethically sound. AI might also omit critical details or insert inaccurate assumptions that could be potentially harmful when dealing with uniquely complex issues (e.g., misclassifying the virulence or toxicity of biological agents) or matters pertaining to vulnerable populations (e.g., psychological risks to participants in studies involving trauma survivors). One might rightly argue that mistakes can, and do, happen without AI adoption. In fact, both the Ellen Roche and Oklahoma Melanoma Vaccine Trial tragedies happened long before the advent of AI tools. Nevertheless, this should not, and cannot justify adopting tools that introduce *more* or *different* types of errors. Furthermore, we should not forget that human-errors happen within a broader ethical, professional, and cultural framework, where norms, responsibilities, and accountabilities exist. AI tools, by contrast, lacks grounding for inclusion as an entity with legal and moral capacity (Resnik and Hosseini 2024), and relying on them introduces additional risks. Let’s take a well-established technical limitation of AI tools, namely the black box problem (i.e., our inability to understand how AI produced a certain outcome) as an example. To accurately understand why an AI produced an incorrect output, and to prevent similar incidents in the future, one would need to probe deeply into a system and examine not only the code but also the weightings attached to inputs and the mathematical computations produced from those inputs. However, this is currently not possible to do. Another concern is AI’s tendency to create fake but seemingly accurate content, commonly referred to as “hallucinations” (although such terminology is inaccurate as AIs lack mental states) (Resnik and Hosseini 2024). Despite these limitations, researchers are currently allowed to utilize AI tools to prepare applications for the Three I’s without much guidance. The same goes about the Three I’s reviewers.

Third, the responsibility problem is not solved by being transparent about AI use in preparing or reviewing the Three I’s applications. When preparing manuscripts for submission to peer-reviewed journals, disclosing AI involvement is sufficient, because authors have the ultimate responsibility for the content (Resnik 2024). However, the Three I’s applications are meant to reflect a research team’s ethical planning, understanding of the applicable moral nuances and risk mitigation plans. Indeed, delegating the writing process to an AI allows researchers to be less knowledgeable about the ethical dimensions of their work. In terms of using AI in reviews, even if AI is only used for triage, and this is transparently disclosed to the panel members, it could possibly affect the views of human reviewers and result in deskilling, i.e., reliance on AI leading to a decline in human intellectual capabilities (Hosseini, Murad, and Resnik 2026). Rather than fostering ethical engagement and debate about proposals, AI-generated reviews may lead to over-reliance on superficial summaries and deter humans from meaningful and thorough engagement with submitted applications, ultimately resulting in a diffusion of responsibilities and accountabilities. Reviewing applications requires highly context-sensitive ethical judgment, including conducting risk-benefit analyses, evaluating the appropriateness of documentation (such as the informed consent form), and identifying subtle kinds of vulnerability or undue influence. AI tools of today cannot make value-based ethical judgments rooted in human empathy, cultural context, and institutional standards.

## Discussion and conclusion

In the 20th century, ethical planning and oversight were integrated into research operations to prevent catastrophes and harmful practices, and to ensure that research aligns with safety standards, ethical and societal norms, and legal mandates (Resnik 2024). To save time and costs, researchers and institutions might be tempted to use AI to prepare and review Three I’s applications. However, the challenges and risks outlined in this paper show that AI use is prone to substantial risks. Accordingly, even in cases where risks are minimal, AI can only be complementary to human skills, and should not replace humans or make important or the final decision.

### Human in the loop is not enough

One can argue that outsourcing ethical planning and oversight to AI represents an abdication of our moral responsibilities and could set the scene for unforeseen future harms. It is for this reason that whenever AI is integrated into research workflows, humans must not only remain in the loop, but also in charge so that they can be held accountable. This is to ensure that AI-assisted decisions consider factors such as sensitivities of the community, institutional environment or risk tolerance, and whether PIs can adequately supervise the research personnel to safely perform the research as described in an application. However, a key concern about using AI with humans in the loop is that human involvement does not always guarantee quality or safety. For example, AI-assisted peer reviewed papers that were later retracted (Retraction Watch 2025; Pearson 2024) have included numerous humans in the loop (i.e., authors, editor in chief, handling editor, peer reviewers, and copyeditors) who all failed to prevent the publication of erroneous papers. Especially, if humans are not incentivized or are inadequately trained, or when AI systems act autonomously, the presence of a human does not necessarily help (Hosseini, Murad, and Resnik 2026). It is for this reason that certain tasks should never be delegated to AI tools, and it is up to experts in each domain to determine which tasks in their context are so sensitive that they can never be outsourced to AI.

### AI can exacerbate existing challenges in the Three I’s

Institutions often rely on checklist-driven Three I’s reviews of protocols, sometimes using off-the-shelf, third-party-produced toolkits. Some may argue that, if checklist users do not fully grasp the reasoning behind the decisions their tools help them reach, why should we be worried about AI causing deskilling? We believe that AI could worsen these problems by further disconnecting researchers and reviewers from ethical reasoning. This is not to say that AI should be excluded from the process altogether, but it underscores the importance of distinguishing between appropriate and inappropriate uses.

Moreover, much evidence exists to suggest the AI-free review processes have already produced inconsistent quality both within and between reviewing institutions (Binik and Hey 2019; Millum and Menikoff 2010). Therefore, employing AI *after* humans have completed their review could provide some assistance to determine if decisions are consistent, to surface any overlooked ethical issues, or to assess whether relevant arguments are overlooked. In such contexts, AI tools could be helpful, but only if they are validated and locally hosted by an organization like a university, as opposed to an off-the-shelf, commercially available tool.

Although it is true that using AI could enhance productivity and efficiency of researchers, one can argue that if further integration of AI into various research and oversight tasks would decrease the overall quality and integrity of science, the risks outweigh the benefits. One such risk is the erosion of societies’ trust in science. It is reasonable to argue that inappropriate and irresponsible use of AI would erode this trust, and harm not only the future of science but also the well-being of individuals/communities who may turn to nonscientific solutions to address health, social, environmental, and other issues.

### Enforcement gaps and their implications

While restricting the use of AI in preparing and reviewing Three I’s applications might be extremely difficult to enforce (given the growing availability of AI tools for preparing and reviewing these applications), providing clear guidance is essential to help researchers and reviewers understand appropriate and inappropriate uses. Clarifying and explaining the difference between using AI in writing or reviewing a manuscript, versus in completing a Three I’s application could help researchers understand the involved risks and encourage adherence. Since some AI use cases might be undetectable, enforcement will remain challenging though. Nevertheless, this should not dissuade the community from attempting to develop and promote best practices. For example, mandating that AI outputs contain embedded watermarks could allow for better tracking and auditing. When investigators and/or authors disclose their use of AI tools, the AI outputs could be uploaded as supplementary materials to the application to enhance provenance and traceability. Perhaps such uploads would be required when AI contributions extend beyond a minimum threshold of defining terms, grammar checks, or refinements of readability.

In terms of using AI in review of the Three I’s applications, although there is currently no evidence that AI has been used as a voting member, eradicating future confusion is helpful: Federal regulations and guidelines pertinent to each committee could specifically suggest that the quorum requirement or voting cannot be satisfied by non-human entities like AI, because adding AI to the quorum would imply that it is a sentient person – which it clearly is not. This would also ensure difficult and nuanced decisions regarding research ethics are ultimately made by humans, rather than computer algorithms. One ideal scenario is the creation of regulations that allow AI to serve as a supporting tool to enhance committee review while preventing it from issuing final determinations. Such regulations provide transparency about the role of AI to assuage public concerns about ethical standards and the quality of scientific outcomes, while ultimately leading to higher-quality reviews.

### AI in the Three I’s: The road ahead

Using AI in preparing or reviewing Three I’s submissions raises serious ethical and procedural questions that must be addressed by research institutions as well as organizations such as the NIH, the Office for Human Research Protections (OHRP), the Office of Science Policy (OSP), the Association for the Accreditation of Human Research Protection Programs (AAHRPP), and Public Responsibility in Medicine and Research (PRIM&R).

When developing policies, some questions to consider may include:

- Can AI be used to prepare the Three I’s submissions? If so, can it be relied upon for the core elements of these documents, particularly sections addressing risks, potential adverse events, harms, mitigation strategies, and ethical justifications where expert judgment and contextual nuance are indispensable?
- How does using AI to prepare Three I’s submission affect researchers’ understanding of ethical requirements, and their behavior when facing ethical issues in practice?
- What AI uses in preparing or reviewing Three I’s submissions should be disclosed and where in a submission or review report should disclosure happen?
- In cases where AI use in preparing an application is disclosed, how should review panels account for this information? Does such disclosure create new responsibilities for reviewers or necessitate additional verification steps?
- To what extent can or should review responsibilities be delegated to AI? More specifically, what review tasks can be delegated to AI and at what stage in the review process?
- Where is the line between efficient support and over-reliance on automation, that may result in deskilling reviewers, and/or compromising accountabilities in the context of Three I’s?

In terms of using AI in the Three I’s reviews, we believe that AI should under no circumstances be utilized to make the final committee or reviewer decision. Rather, it should only serve as a tool to enhance and support the decision-making of human members. Given that there are various forms of ethical oversight and submission types (e.g., expedited, exempt, and full board review), it is essential that review committee policies target unique categories with specific requirements. For example, one possibility might be that once a human determines that an application only requires expedited review, an AI tool could be used for quality control to verify that all the required components supporting such a determination are present and correct.
